# Editorial: Biobanks as Essential Tools for Translational Research: The Belgian Landscape

**DOI:** 10.3389/fmed.2020.00378

**Published:** 2020-07-28

**Authors:** Annelies Debucquoy, Loes Linsen, Veronique T'Joen, Laurent Dollé, Sofie Bekaert

**Affiliations:** ^1^BBMRI.be, Belgian Cancer Registry, Brussels, Belgium; ^2^AC Biobanking, University Hospitals Leuven, Leuven, Belgium; ^3^ESBB, Brussels, Belgium; ^4^Biothèque Wallonie Bruxelles, Brussels, Belgium; ^5^Department of Public Health and Primary Care, Faculty of Medicine and Health Sciences, Gent, Belgium; ^6^Manager Translational Program VIB, Gent, Belgium

**Keywords:** biobank, samples, data, biobank network, BBMRI

BBMRI.be (1), the Belgian biobank network and Belgian National Node of the European biobank infrastructure BBMRI-ERIC (2), was set up in order to support the ever-increasing need for human biospecimen samples for research guaranteeing quality control, access, transparency, and interconnectedness of biobanks (3). The BBMRI.be network was initiated by uniting the three existing Belgian network biobank initiatives i.e., Belgian Virtual Tumourbank (BVT) (4) project assigned to the Belgian Cancer Registry, Biothèque Wallonie-Bruxelles (BWB) (5) and the Flemish Biobank Network (CMI). From 2013 to 2019, BBMRI.be has matured into a solid partner network of 16 biobanks in Belgium and has proven to reach out to a broader community beyond the founding partners. From 2019 onwards, BBMRI.be invites all Belgian biobanks with translational research potential as well as biobank user organizations that are seeking structural research collaborations to join the BBMRI.be network. The strong representation of several members of BBMRI.be in working groups of local, regional, and national decision-making organizations covering ethical, legal and other aspects of biobanking [FAMPH (6), BAREC (7), VLIR (8), BVT (4), BWB (5), NBN (9)…] as well as the active participation in international biobank networks and associations [ESBB (10), ISBER (11), BBMRI-ERIC (2), 3C-R (12), ISO (13) …] and regional health/life Sciences Clusters [BioWin (14), LifeTech Brussels (15), Flanders.bio (16)] assures a good cross-fertilization on all levels and pushes forward the development of the biobank community.

With the current Research Topic, we focus on the challenges local biobanks and biobank networks are facing along the road toward implementation and sustainability and how these can be overcome. We also share some success stories illustrating how, over a decade, the BBMRI.be biobank network has managed to build strong cornerstones and become a fertile substrate for human biospecimen samples management and access for translational research purposes.

First, we highlight the new processes and strategies implemented by the Belgian biobanks to optimize their biobank activities in light of new quality standards and changing national laws legislation. The first manuscript describes the evolution of the University Biobank Limburg (UBiLim) from an archival sample collection into a federated biobank structure, supporting translational research, dissecting the major challenges at each stage (Linsen et al.). A campus-wide cell line dataset was developed in the biobank of the Ghent University Hospital (T'Joen et al.), to enhance cell line data quality and its usability in the translational research community. The third paper describes the extensive setup and validation process of two automated sample storage and retrieval systems at the UZ KU Leuven biobank (Linsen et al.), detailing the problems encountered and the efforts needed to obtain successful implementation.

As already illustrated above, quality in biobanking is crucial for the long-term sustainability of the biobank and for the reproducibility of the downstream research. This message is further emphasized by work from the Quality Working Group of BBMRI.be which assessed and demonstrated a solid quality approach and mindset in the Belgian biobanks (Linsen et al.). Another illustration thereof is depicted by Craciun et al., with a hands-on example on how the quality of samples can be assured by implementing quality control schemes in the biobank, either by internal quality control test or by participating to external quality test such as the ISBER Proficiency Testing.

The BBMRI.be biobank network hosts a treasure of very valuable collections, a flavor of which is presented in this Research Topic, where we share some success stories from collections stored in our biobanks.

The Belgian Virtual tumorbank, described by Vande Loock et al., connects the tumor biobanks from 11 Belgian hospitals. While all biobanks store the residual human tumor samples locally, the data is centrally registered at the Belgian Cancer Registry and available for researchers in the field of oncology. The manuscript describes the setup of the virtual network, the quality checks performed on the data and gives an overview of the samples and associated data available for research.

The Inflammatory bowel disease collection (Cleynen et al.) was built up as a collaboration between three Belgian IBD centers (University Hospitals Brussels, Ghent and Leuven) and has evolved over the years into a valuable source of material from patients with IBD and normal controls. The paper details the setup of this collection and demonstrates its added value by sharing some success stories that were obtained with samples and data collected within this framework.

The Cardiogeneticsbank@UZA biobank (Alaerts et al.) and the collection on viral hepatitis (Ho et al.) are both integrated in the biobank of Antwerp. The Cardiogenetics biobank collected samples and data of patients with a cardiogenetic disorder. In the manuscript, several research projects are described to illustrate the potential of these valuable collections and the prospects for future research. The Viral Hepatitis collection is a unique collection that was established by collecting samples from hepatitis patients collected both in-hospital and during community outreach screenings. The publication describes the setup and associated challenges of both the in-hospital as community collections, the samples that were obtained and some research results that were acquired with these samples.

Van den Heuvel et al. focus on the VITO biobank and illustrate the potential of a population biobank. This biobank, with about 70.000 biological samples from the general population in Flanders, was set up to answer research questions related to health and environment. Samples were collected within different human biomonitoring studies and are linked with extended data on the lifestyle, environment, and health status of the donor.

The manuscripts assembled in this Research Topic clearly illustrate the value of the Belgian biobanks as catalyst for translational and clinical research. However, biobanks are to the best of their means the custodians of the most precious human biospecimen samples donated by patients and healthy volunteers. The input of these important stakeholders should therefore be implemented into the biobanking process. The manuscript of Broes et al., in which patients were questioned about their view on re-use of clinical trial samples and data is an excellent example of how sharing knowledge and engaging with patients, might help to push forward the biobank community.

This Research Topic gives an impression of the numerous research opportunities with human biospecimen samples and data from the Belgian biobanks and illustrates how biobanks are an essential tool for translational research.

## Author Contributions

All authors listed have made a substantial, direct and intellectual contribution to the work, and approved it for publication.

## Conflict of Interest

The authors declare that the research was conducted in the absence of any commercial or financial relationships that could be construed as a potential conflict of interest.
